# Critical parameters and procedures for anaerobic cultivation of yeasts in bioreactors and anaerobic chambers

**DOI:** 10.1093/femsyr/foab035

**Published:** 2021-06-08

**Authors:** Christiaan Mooiman, Jonna Bouwknegt, Wijb J C Dekker, Sanne J Wiersma, Raúl A Ortiz-Merino, Erik de Hulster, Jack T Pronk

**Affiliations:** Department of Biotechnology, Delft University of Technology, Van der Maasweg 9, 2629 HZ Delft, The Netherlands; Department of Biotechnology, Delft University of Technology, Van der Maasweg 9, 2629 HZ Delft, The Netherlands; Department of Biotechnology, Delft University of Technology, Van der Maasweg 9, 2629 HZ Delft, The Netherlands; Department of Biotechnology, Delft University of Technology, Van der Maasweg 9, 2629 HZ Delft, The Netherlands; Department of Biotechnology, Delft University of Technology, Van der Maasweg 9, 2629 HZ Delft, The Netherlands; Department of Biotechnology, Delft University of Technology, Van der Maasweg 9, 2629 HZ Delft, The Netherlands; Department of Biotechnology, Delft University of Technology, Van der Maasweg 9, 2629 HZ Delft, The Netherlands

**Keywords:** anaerobic, cultivation, oxygen requirements, saccharomyces, nonconventional yeast

## Abstract

All known facultatively fermentative yeasts require molecular oxygen for growth. Only in a small number of yeast species, these requirements can be circumvented by supplementation of known anaerobic growth factors such as nicotinate, sterols and unsaturated fatty acids. Biosynthetic oxygen requirements of yeasts are typically small and, unless extensive precautions are taken to minimize inadvertent entry of trace amounts of oxygen, easily go unnoticed in small-scale laboratory cultivation systems. This paper discusses critical points in the design of anaerobic yeast cultivation experiments in anaerobic chambers and laboratory bioreactors. Serial transfer or continuous cultivation to dilute growth factors present in anaerobically pre-grown inocula, systematic inclusion of control strains and minimizing the impact of oxygen diffusion through tubing are identified as key elements in experimental design. Basic protocols are presented for anaerobic-chamber and bioreactor experiments.

## Teaser

An overview of the challenges in anaerobic cultivation systems for yeast, and current best practices using anaerobic chambers and bioreactors.

## INTRODUCTION

When grown under oxygen-limited conditions, the large majority of currently known yeast species at least partially ferment glucose to ethanol and carbon dioxide (Barnett, Payne and Yarrow 1983; van Dijken *et al*. 1986; Visser *et al*. 1990; Merico *et al*. 2007). The ability of these facultatively fermentative yeasts to generate ATP by substrate-level phosphorylation does not, however, imply they are all able to grow in the complete absence of oxygen. Instead, with few exceptions, even yeast species that vigorously ferment glucose under oxygen-limited conditions cannot sustain growth under strictly anaerobic conditions. This inability reflects small and often undefined oxygen requirements, which are generally attributed to a direct requirement of biosynthetic reactions for molecular oxygen and/or coupling of reactions in biosynthesis to the mitochondrial respiratory chain (Visser *et al*. 1990; Panozzo *et al*. 2002; Snoek and Steensma 2006; Summons *et al*. 2006; Martin, Oh and Jiang 2007; Wiersma *et al*. 2020).

The model yeast *Saccharomyces cerevisiae* is one of few yeasts capable of fast anaerobic growth on synthetic mineral media supplemented with a fermentable sugar, a defined set of B-type vitamins, a source of sterols and unsaturated fatty acids (UFAs; Wheeler and Rose 1973; Bulder and Reinink 1974; Verduyn *et al*. 1990, 1992; Fekete, Ganzler and Fekete 2010; Perli *et al*. 2020a). The requirement of anaerobic yeast cultures for sterols and UFAs, which are frequently referred to as ‘anaerobic growth factors’, is due to the use of molecular oxygen in sterol biosynthesis and fatty acyl-CoA desaturation, respectively (Andreasen and Stier 1953a,b; Summons *et al*. 2006; Martin, Oh and Jiang 2007; Liu *et al*. 2019). Although the requirement of anaerobic *S. cerevisiae* cultures for UFAs is not absolute, anaerobic growth in the absence of UFA supplementation is slow (Dekker *et al*. 2019). The maximum specific growth rate of *S. cerevisiae* in sterol- and UFA-supplemented, glucose-grown anaerobic batch cultures is typically only about 25% lower than in corresponding aerobic cultures (Verduyn *et al*. 1990). This fast anaerobic growth of *Saccharomyces* yeasts is exceptional among yeasts (Visser *et al*. 1990; Merico *et al*. 2007) and essential for their large-scale industrial application in brewing, wine fermentation and bioethanol production (Casey, Magnus and Ingledew 1984; Mauricio, Milla and Ortega 1998; Holm Hansen *et al*. 2001; Depraetere *et al*. 2008; Parapouli *et al*. 2020). In industrial settings, large-scale fermentation processes are often preceded by an aerobic pre-cultivation phase or, alternatively, by a brief phase of intensive aeration, which enables cells to build intracellular storage of sterols and UFAs. In such set-ups, oxygen requirements for sterol and unsaturated-fatty-acid (UFA) synthesis can still negatively affect strain performance during prolonged anaerobic cultivation. For example, premature depletion of sterols and/or UFAs can cause ‘stuck’ brewing and wine fermentations (Munoz and Ingledew 1989; Bisson 1999). In addition, UFAs as well as ergosterol contribute to tolerance of *S. cerevisiae* to ethanol stress (Vanegas *et al*. 2012; Johnston, Moses and Rosser 2020), which is an important factor for intensification of yeast-based processes for ethanol production.

The magnitude and molecular basis of the oxygen requirements of most yeasts other than *S. cerevisiae*, remain to be fully elucidated (Snoek and Steensma 2006; Merico *et al*. 2009). Analyzing and understanding oxygen requirements of facultatively fermentative ‘non-conventional’ yeasts can contribute to our comprehension of the roles of molecular oxygen in eukaryotic metabolism. In addition, such knowledge is essential for designing metabolic engineering strategies to enable application of non-conventional yeasts with industrially relevant traits, such as thermotolerance and inhibitor tolerance, in large-scale anaerobic processes (Lacerda, Oh and Eckert 2020; Sun and Alper 2020; Thorwall *et al*. 2020).

Oxygen requirements for synthesis of key cellular components in *S. cerevisiae* can be estimated from the stoichiometry of biosynthetic reactions and reported data on biomass composition (Table 1). Although biomass composition may vary among strains and depend on cultivation conditions, such an analysis readily identifies synthesis of sterols and UFAs as the major oxygen-requiring biosynthetic processes. However, their combined oxygen requirement of approximately 0.1 mmol O_2_/(g biomass) (Table 1) requires an oxygen consumption rate of only 0.16 μmol (g biomass)/min to sustain a specific growth rate of 0.10/h (doubling time of 6.9 h) that is used as a reference in many yeast chemostat studies (Tai *et al*. 2005; van Eunen *et al*. 2010; Papini *et al*. 2012).

**Table 1.** Estimated oxygen requirements for biosynthesis of ergosterol, unsaturated fatty acids and other components of yeast biomass.

Biomass component *Medium component*	O_2_ stoichiometry of biosynthesis (mol O_2_/mol)	Content in *S. cerevisiae* biomass (µmol/g biomass)	*S. cerevisiae* strain and growth conditions	Reference	O_2_ requirement for biosynthesis (µmol/g biomass)
Ergosterol *Ergosterol*	12	4.3	CBS2806, glucose-limited chemostat	Arneborg, Høy and Jørgensen (1995)	30–52
		2.6	CEN.PK113-7D, glucose-limited chemostat	da Costa *et al*. (2019)	
		3.9	CEN.PK113-7D, batch culture	Wiersma *et al*. (2020)	
UFA^a^*Tween 80*	1	21 (1.6 C_16:1_, 19.4 C_18:1_)	CBS2806, glucose-limited chemostat	Arneborg, Høy and Jørgensen (1995)	21–103
		103 (44 C_16:1_, 59 C_18:1_)	CEN.PK113-7D, glucose-limited chemostat	da Costa *et al*. (2019)	
		58 (1.2 C_16:1_, 57 C_18:1_)	CEN.PK113-7D, batch culture	Wiersma *et al*. (2020)	
Pyridine nucleotides *Nicotinate*^b^	3	3.8	CEN.PK113-7D, glucose limited chemostat	Seifar *et al*. (2013)	6.3–14
		4.5	Strain 210NG, aerobic ethanol-stat vitamin fed-batch	Paalme *et al*. (2014)	
		2.1–3.9	CEN.PK113-7D, glucose-limited accelerostat (NAD^+^/NADH only)	Bekers, Heijnen and Gulik (2015)	
Biotin *Biotin*	∼1^c^	0.002–0.009	Industrially produced yeast	Suomalainen and Keränen (1963)	∼0.002–0.009
		0.002–0.008	Strain 1403–7A, aerobic uptake assay	Kosugi *et al*. (2000)	
Coenzyme A *Pantothenate*^d^	1	0.43	Glucose limited chemostat (sum of CoA and acetyl-CoA)	Seifar *et al*. (2013)	∼0.4
		∼0.38	CEN.PK2-1C, aerobic shake flask (only acetyl-CoA)	Liu, Zhang and Jiang (2017)	
Thiamine *Thiamine*	∼4^c^	0.025–0.22	Strain 210NG, aerobic ethanol-stat vitamin fed-batch	Paalme *et al*. (2014)	∼0.0022–0.22
		0.0022–0.0029	Brewing strain #1007, static and shaken wort cultures	Hucker, Wakeling and Vriesekoop (2016)	
Pyrimidines^e^*Uracil*	0.5	179	Strain 306, oxygen-limited continuous cultures	Oura (1972)	56–90
		111	Biomass equation in genome-scale model	Förster *et al*. (2003)	

Unless otherwise indicated, data are based on reported biomass compositions of *S. cerevisiae* strains in anaerobic experiments with supplementation of Tween 80 (polyoxyethylene sorbitan monooleate), ergosterol and a selection of B-type vitamins.

a Values are given as: Total UFA's (palmitoleate, C_16:1_/oleate, C_18:1_).

b Nicotinate is a precursor for oxygen-independent synthesis of nicotinamide adenine dinucleotides (NAD^+^/NADP^+^).

c Oxygen-dependent reactions involved in thiamin and biotin biosynthesis by yeasts have not been fully resolved, and the indicated stoichiometries are estimates. For thiamine, the requirement of 3 moles of oxygen for the synthesis of the required NAD^+^ moiety is incorporated.

d Pantothenic acid is a precursor for oxygen-independent synthesis of coenzyme A.

e *Saccharomyces cerevisiae* strains do not require oxygen for pyrimidine biosynthesis. Estimated oxygen requirements refer to yeasts in which pyrimidine biosynthesis depends on a respiratory-chain-coupled dihydroorotate dehydrogenase, assuming a DNA and RNA content equal to that of *S. cerevisiae*.

In *S. cerevisiae*, biosynthesis of several key cofactors and their precursors requires oxygen (Table 1). In the *de novo* synthesis of the pyridine-nucleotide cofactors NAD^+^ and NADP^+^, the reactions catalysed by Bna2, Bna4 and Bna1 each require one mole of oxygen (Panozzo *et al*. 2002). Similarly, Fms1 catalyses an oxygen-dependent reaction in pantothenate biosynthesis (White, Gunyuzlu and Toyn 2001), while synthesis of thiamine requires at least three moles of oxygen because NAD^+^ acts as a precursor. In addition, *S. cerevisiae* has an incompletely understood oxygen requirement for synthesis of the thiamine precursor hydroxymethylpyrimidine (Wightman and Meacock 2003). Recent research indicates that also the first, unresolved step of biotin biosynthesis in yeasts, catalysed by Bio1 orthologs, is oxygen dependent (Wronska *et al*. 2020). Although heme biosynthesis is oxygen dependent, heme-containing proteins in *S. cerevisiae* are strongly associated with aerobic metabolism. Since, moreover, sulfite reductase contains siroheme rather than heme (Tripathy, Sherameti and Oelmüller 2010), anaerobic cultivation of *S. cerevisiae* does not require heme supplementation.

With the exception of pyridine-nucleotide synthesis, oxygen requirements for cofactor biosynthesis are at least two orders of magnitude lower than those for sterol and UFA biosynthesis (Table 1) and, in laboratory studies with synthetic media, they are usually masked by the routine inclusion of a mix of B-type vitamins (Perli *et al*. 2020b).

In most non-*Saccharomyces* yeasts, pyrimidine metabolism depends on a mitochondrial, respiratory-chain-coupled dihydroorotate dehydrogenase (Wolfe 2006; Riley *et al*. 2016) and therefore contributes a biosynthetic oxygen requirement similar in magnitude to that for sterol and UFA synthesis (Table 1). Pyrimidine biosynthesis in *S. cerevisiae* does not require oxygen, because its soluble cytosolic dihydroorotate dehydrogenase (Ura1) uses fumarate as electron acceptor (Nagy, Lacroute and Thomas 1992; Gojković *et al*. 2004).

Studies on the quantification and elucidation of oxygen requirements of yeasts, as well as physiological studies on the effects of severe oxygen limitation, require the option to reduce oxygen entry into yeast cultivation systems to extremely low levels (Visser *et al*. 1990; da Costa *et al*. 2018; Dekker *et al*. 2019; Wiersma *et al*. 2020). Here, we focus on two cultivation systems that are commonly used in such anaerobic growth studies with yeasts. Anaerobic chambers, filled with a hydrogen-containing atmosphere and equipped with a Pd catalyst to remove traces of oxygen, are often used for anaerobic batch cultivation of yeasts in shake flasks or on plates (Thomas, Hynes and Ingledew 1998; Kozak *et al*. 2014; Madeira-Jr and Gombert 2018). Laboratory bioreactors are popular systems to perform controlled batch, fed-batch or continuous cultivation of yeasts under anaerobic conditions. Closed systems such as anaerobic jars for cultivation on agar plates, serum flasks and Hungate tubes will not be discussed in view of their limited applicability for quantitative analysis of growth, physiology and gene expression.

Vessels and lids of bioreactors are typically made of oxygen-impermeable materials such as glass and/or stainless steel. Their area-to-volume ratio (A/V), which is sometimes mentioned as a key factor in oxygen diffusion (Simpson and Sastry 2013; da Costa *et al*. 2018), is therefore not in itself a key factor in oxygen leakage. Instead, synthetic tubing, rings and seals, as well as sensors and sampling ports, are among the key potential entry points for oxygen. Since the surface area of these sensitive points (A_S_) does not scale with reactor volume, A_S_/V is orders of magnitude lower in large-scale industrial bioreactors than in bench-top laboratory set-ups (Ju and Chase 1992). When bioreactors are operated as fed-batch or chemostat cultures, additional precautions are needed to prevent oxygen entry via the medium feed (Visser *et al*. 1990).

Experimental challenges involved in preventing oxygen leakage into laboratory cultures have contributed to conflicting reports on the ability of yeast species and strains to grow anaerobically (Macy and Miller 1983; Thomas, Hynes and Ingledew 1998; Møller, Olsson and Piškur 2001; Snoek and Steensma 2006; Wilkins *et al*. 2008; Hughes *et al*. 2013; da Costa *et al*. 2019). Although these challenges are frequently mentioned in the literature (Visser *et al*. 1990; Rodrigues *et al*. 2001; da Costa *et al*. 2018), we are not aware of publications that combine a discussion of critical points in design of anaerobic yeast cultivation experiments with laboratory protocols. Based on experience in our laboratory spanning three decades (Verduyn *et al*. 1990; Visser *et al*. 1990; Dekker *et al*. 2019; Wiersma *et al*. 2020), this paper aims to discuss pitfalls and challenges and share our current protocols for anaerobic cultivation of yeasts in anaerobic chambers and bioreactors.

## INTRACELLULAR RESERVES AND CARRY-OVER OF ANAEROBIC GROWTH FACTORS

Many yeast species accumulate lipids, including UFAs and sterols, in lipid droplets during aerobic growth on glucose (Clausen *et al*. 1974; Rajakumari, Grillitsch and Daum 2008; Kohlwein 2010). Toxic effects of intracellular free fatty acids and sterols are prevented by storage as nonpolar steryl esters (SE) and triacylglycerol (TAG) lipids (Eisenberg and Büttner 2014). Lipid droplet synthesis from extracellular sources of sterols and UFAs has also been observed in heme-deficient cells (Spanova *et al*. 2010, 2012) and under anaerobic conditions (Valachovic 2001; Boender *et al*. 2011). Such intracellular stores of lipids can be mobilized to supply sources for membrane synthesis (Meyers, Weiskittel and Dalhaimer 2017). Redistribution of the released UFAs and sterols over dividing yeast cells may therefore enable multiple generations upon transfer to strictly anaerobic conditions, even if sterols and UFAs are not included in growth media. This phenomenon is applied in industrial brewing, in which a brief aeration phase enables the generation of endogenous sterol and UFA reserves, which then support growth and fermentative capacity during the subsequent anaerobic fermentation process (Casey, Magnus and Ingledew 1984).

**Figure 1.**
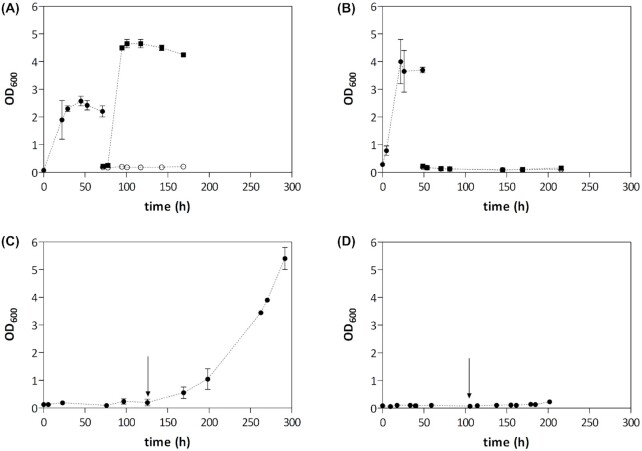
Representative growth profiles of four facultatively fermentative yeast species in standardized anaerobic chamber experiments. Anaerobic-chamber experiments were performed as described in Protocol 1. The yeasts *S. cerevisiae***(A)**, *K. marxianus***(B)**, *B. bruxellensis***(C)** and *Tetrapisispora phaffii***(D)** were grown in 50-mL shake-flasks containing 40 mL synthetic medium with urea as nitrogen source (SMU; Luttik *et al*. 2000), with supplements as indicated below. An anaerobic pre-culture (closed circles) without ergosterol or Tween 80, supplemented with 50  g/L glucose, was inoculated within the anaerobic chamber with an inoculum that had been grown aerobically on SMU with 20  g/L glucose until late exponential phase. When growth had occurred in this anaerobic pre-culture (A and B), and no further increase of the optical density was observed, aliquots were transferred to flasks with fresh SMU with 20  g/L glucose, either containing no anaerobic growth factors (open circles), or both Tween 80 and ergosterol (closed squares). When no growth was observed in the anaerobic pre-cultures for at least 100 h (C and D), a Tween 80 and ergosterol pulse was administered (indicated by arrows) and growth was further monitored. Data are represented as averages and mean deviation of two independent biological replicate cultures for each strain.

‘Carry-over’ of extracellular and/or intracellular reserves of anaerobic growth factors or their precursors may obscure biosynthetic oxygen requirements of yeasts in laboratory studies (Macy and Miller 1983; Thomas, Hynes and Ingledew 1998; Dekker *et al*. 2019). For example, significant growth is observed upon inoculation of anaerobic shake-flask cultures of *S. cerevisiae* on glucose synthetic media without sterols or UFAs with aerobically pregrown cells (Fig. 1). Increasing the glucose concentration in the anaerobic cultures can help to deplete intracellular reserves before glucose is completely consumed. In such experiments depletion of reserves is reflected by the absence of growth upon transfer of cells to a subsequent anaerobic culture on sterol- and UFA-free medium (Fig. 1). The extent to which yeast strains or species grow during such a first cycle of anaerobic growth designed to deplete stores of anaerobic growth factors has no predictive value for their ability to subsequently grow in Tween 80 and sterol-supplemented medium (Fig. 1). For example, *Brettanomyces* (*Dekkera) bruxellensis* cannot sustain anaerobic growth on intracellular reserves, but initiates growth upon addition of Tween 80 and ergosterol. Conversely, *Kluyveromyces marxianus* grows anaerobically in the growth-factor depletion culture, but not upon transfer to fresh medium with or without these growth factors. This pattern may reflect additional nutritional requirements for anaerobic growth and, in *K. marxianus*, has recently been attributed to absence of a functional sterol uptake system (Dekker *et al*. 2021). These observations underline the necessity, irrespective of the cultivation system, to include a dedicated anaerobic pre-cultivation step to deplete intracellular growth factors in batch-cultivation studies on anaerobic nutritional requirements of yeasts.

## MONITORING ANAEROBICITY OF YEAST CULTURES

**Figure 2.**
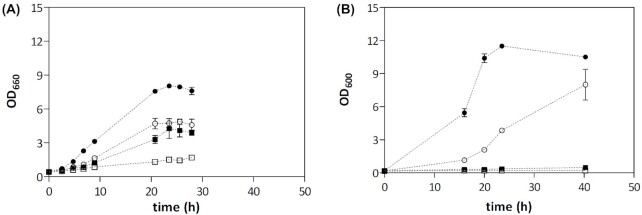
Use of the air lock of an anaerobic chamber as main source of oxygen contamination. Anaerobic-chamber experiments were performed as described in Protocol 1. *S. cerevisiae* CEN.PK113-7D was grown in 50-mL shake-flasks containing 40 mL synthetic medium with urea as nitrogen source (SMU; Luttik *et al*. 2000), with supplements as indicated below. An anaerobic pre-culture without ergosterol or Tween 80, supplemented with 50  g/L glucose, was inoculated within the anaerobic chamber with an inoculum from an exponentially growing aerobic culture on SMU with 20  g/L glucose. After the optical density increase in the pre-culture levelled off, aliquots were transferred to flasks with fresh SMU with 20  g/L glucose, supplemented with Tween 80 and ergosterol (closed circles), ergosterol only (open circles), Tween 80 only (closed squares) or SMU without these anaerobic growth factors (open squares). **(A)** Optical density measurements at 660 nm were performed outside the anaerobic chamber, requiring frequent use of the air lock. **(B)** Optical density was measured within the anaerobic chamber at a wavelength of 600 nm (different wavelength due to use of dedicated fixed-wavelength spectrophotometer in anaerobic chamber). This decreased the need to open the doors of the air lock. Data are represented as averages and mean deviation of two independent biological replicate cultures for each condition.

Facultatively fermentative yeasts that cannot grow under strictly anaerobic conditions, typically show fast growth at dissolved-oxygen concentrations that are below the detection level of the polarographic oxygen probes that are commonly used in microbial cultures (van Dijken *et al*. 1986; Merico *et al*. 2009). Measurement of dissolved-oxygen concentrations is, therefore, not a reliable way to assess culture anaerobicity. In anaerobic chambers, indicator cultures that require small amounts of oxygen for growth can be used as a control for oxygen contamination (Snoek and Steensma 2006). For example, *Kluyveromyces lactis* cannot grow on synthetic glucose medium with Tween 80 and ergosterol under strictly anaerobic conditions, but shows fast fermentative growth in oxygen-limited cultures (Merico *et al*. 2009). In our experience, cultures of a wild-type *S. cerevisiae* strain on synthetic glucose medium lacking Tween 80 and ergosterol provides an even more sensitive detection of oxygen leakage (Fig. 2).

In bioreactor cultures, indicator strains cannot be in the same anaerobic compartment as the strain of interest. As outlined in several studies on anaerobic yeast cultivation, it is virtually impossible to eliminate oxygen leakage in bench-top bioreactors (Visser *et al*. 1990; da Costa *et al*. 2018; Dekker *et al*. 2019; Wiersma *et al*. 2020). In studies on oxygen-independent synthesis of (presumed) anaerobic growth factors by wild-type and engineered yeast strains, mutants in which relevant oxygen-dependent reactions have been eliminated therefore provide essential negative controls. For example, sterol-independent anaerobic growth of an *S. cerevisiae* strain expressing a eukaryotic squalene-tetrahymanol cyclase was confirmed by deleting *ERG1* (which encodes an essential enzyme in sterol synthesis (Wiersma *et al*. 2020)).

## ANAEROBIC CHAMBERS

Anaerobic chambers are designed to provide gas-tight, near oxygen-free interior workspaces, in which experiments can be performed by using arm-length gloves made of materials, that are resistant to oxygen diffusion, such as butyl rubber. Materials, including flasks, chemicals and inocula can be transferred to and from the anaerobic workspace via an air lock (‘pass box’). A Pd catalyst, combined with a hydrogen-containing atmosphere, is generally used to remove traces of oxygen from the workspace (Speers, Cologgi and Reguera 2009).

Air locks are designed to remove oxygen before transfer of materials to the anaerobic workspace. However, growth experiments with *S. cerevisiae* in the presence and absence of sterol- and UFA-supplementation indicate that, even when manufacturers’ protocols are strictly followed, use of the air lock can be a significant cause of oxygen entry (Fig. 2). Reducing the void volume of the air lock by inserting inert solid objects helps to reduce oxygen entry (see Protocols). Media should be pre-incubated in the anaerobic air lock and/or in the workspace before inoculation to remove traces of oxygen. Furthermore, experiments should be designed to minimize use of the air lock and, where possible, to synchronize it with catalyst replacement. This minimization implies that routine analyses such as optical density measurements should be performed inside the anaerobic workspace rather than by regular use of the air lock for analyses on external equipment. In view of their restricted options for sampling, sample handling and long-term aseptic operation of cultures, anaerobic chambers are particularly useful for simple, parallel batch-cultivation studies in shake flasks or deep-well plates, for example to compare multiple yeast strains or cultivation conditions.

## BIOREACTORS

Bench-top bioreactors, with working volumes ranging from 0.5 to 5 L, are widely used in quantitative microbial physiology. In contrast to the simple cultivation systems that are generally used in anaerobic chambers, they allow for simultaneous measurement and tight control of multiple process conditions, including pH, temperature, dissolved-oxygen and biomass concentration. For these reasons, laboratory-scale bioreactor cultures are also popular models for design and optimization of large-scale industrial fermentation processes. Bioreactors can be operated in batch, fed-batch or continuous mode which are defined by the medium supply- and broth withdrawal regimes (Verduyn *et al*. 1990; Lee *et al*. 1999; Kallscheuer *et al*. 2019; Wiersma *et al*. 2020).

For anaerobic yeast cultivation in bioreactors, gas with a near zero oxygen content is continuously flushed through the cultures, in most cases in the form of high-purity nitrogen (N6) gas. However, the complexity of laboratory bioreactors makes them prone to permeation of oxygen through seals and tubing, oxygen contamination in gas and liquid flows and/or oxygen leakages through sampling ports, sensors, mass flow controllers and valves. Setting up (near-)anaerobic bioreactor cultures therefore requires great attention for experimental design.

**Figure 3.**
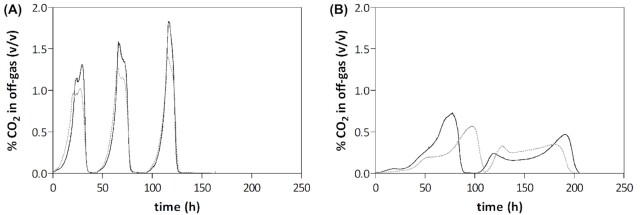
Nitrogen sparging versus headspace supply: impact on growth of *S. cerevisiae* in sequential batch reactors (SBR). Bioreactors were assembled according to Protocol 2. Anaerobic bioreactors were operated in SBR mode, and the CO_2_ content of the off-gas was used to monitor growth of *S. cerevisiae* strain IMX585 (Mans *et al*. 2015). Cultures were grown on synthetic medium with urea as nitrogen source (SMU; Luttik *et al*. 2000). Nitrogen 6.0 HiQ gas (Linde AG, Schiedam, the Netherlands) was supplied to the reactor at 0.5 L/min either by sparging **(A)** or through the reactor headspace **(B)**. When N_2_ was supplied by sparging, an initial anaerobic batch culture on SMU with 25  g/L glucose, lacking ergosterol and Tween 80 was followed by three consecutive SBR cycles on SMU with 20  g/L glucose supplemented with Tween 80 but not with ergosterol. In the cultures to which N _2_ was supplied to the headspace, only two consecutive batch cultures were monitored. Data shown in the figures are from two individual biological replicates for each mode of nitrogen supply, indicated by black and grey lines.

Gas can be supplied to bioreactors either by sparging the stirred liquid phase or by leading gas through the reactor headspace. As bubble formation greatly increases the gas–liquid interface, sparging enables more efficient gas transfer than headspace aeration. In an ideal system, i.e. without any oxygen entry into the reactor, both modes of gas supply should yield the same results. When, instead, inadvertent oxygen entry occurs primarily via the inlet gas, e.g. due to oxygen contamination of high-purity nitrogen (N6) gas, supply through the headspace leads to a lower oxygen transfer to the broth, in which yeast cells maintain a vanishingly low oxygen concentration (van Dijken *et al*. 1986; Merico *et al*. 2009; Dekker *et al*. 2019). Conversely, when oxygen predominantly enters via liquid flows or submerged sampling ports, sparging is preferable. Applying overpressure in the reactor may reduce entry of oxygen through small leaks connected to the headspace. However, at the same time, overpressure will facilitate transfer of any oxygen that enters the reactor as contaminations of the inlet gas flow, as it increases the partial pressure gradient for oxygen transfer to the broth. For our 2-L bioreactor set-ups, we have found that headspace aeration, combined with a small 0.2 bar overpressure to prevent oxygen entry during sampling, results in a lower oxygen availability than sparging (Fig. 3).

Polarographic oxygen electrodes do not detect minor oxygen leaks in growing cultures and can therefore be omitted from anaerobic cultivation set-ups. Probes for pH measurement are often made of porous glass and are not sealed in a gas tight manner. In our anaerobic bioreactor set-ups, we therefore generally accept the absence of active pH control (Dekker *et al*. 2019; Wiersma *et al*. 2020). To prevent the decrease of culture pH caused by ammonium consumption, use of urea as alternative nitrogen source is a straightforward way to avoid excessive acidification (Hensing *et al*. 1995; Luttik *et al*. 2000). Alternatively, a buffering compound can be included in the medium.

Bioreactors are typically connected to a significant length of synthetic tubing to enable addition of liquids and gasses. Tubes connected to the reactor are opened and closed with clamps or valves or, alternatively, inserted in peristaltic pumps. Since permeation through tubing can be a major source of oxygen entry into reactors (Visser *et al*. 1990; da Costa *et al*. 2018), choosing the right material is crucial. In selecting tubing materials, not only oxygen permeability but also factors such as tolerance to autoclaving, resistance to tearing at steel-tubing connections and ruggedness of tubing used in peristaltic pumps need to be considered. For a long time, our group relied on Norprene A-60-G tubing for anaerobic bioreactor set-ups (Verduyn *et al*. 1990). For research on biosynthetic oxygen requirements, we recently changed to Fluran F-5500-A for all gas and liquid tubing, as it has a much lower oxygen permeability than Norprene A-60-G (Table 2). Masterflex C-Flex Ultra has an even lower oxygen permeability to Fluran F-5500-A but in our hands was considerably less resistant to autoclaving, which caused loss of flexibility. In addition, Fluran F-5500-A could also be used in peristaltic pumps, although this requires regular recalibration of pump rates during prolonged operation.

**Table 2.** Characteristics of tubing material for anaerobic bioreactor cultivation. Silicone Peroxide and Norprene A-60-G tubing are commonly used for liquid and gas flows in aerobic and anaerobic laboratory bioreactor cultivation experiments, respectively. Oxygen permeability is expressed in Barrer (10^-10^ cm^3^_STP_·cm/(cm^2^·s·cmHg)); rate of diffusion, at a given pressure, through an area of material with a specified thickness).

Tubing	O_2_ permeability (Barrer)	Autoclavability
Silicone peroxide	4715	++
Norprene A-60-G	200	+++
Fluran F-5500-F	14	+
Nylon	5.4	+
C-Flex ultra	1.1	—

In bioreactors, depletion of intracellular reserves of anaerobic growth factors can be achieved by automated sequential batch-reactor (SBR) cultivation. In SBR set-ups, the reactor is manually or automatically emptied upon reaching a predefined biomass density or CO_2_ output, leaving a small volume of culture broth to act as inoculum after automatic refilling with fresh sterile medium. Alternatively, reactors can be operated as fully continuous (chemostat) cultures. These (semi-) continuous modes of operation require that not only the bioreactors themselves, but also the medium reservoirs are gassed with nitrogen (N5.5).

**Figure 4.**
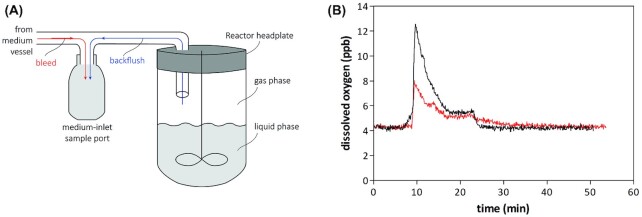
Effect of bleed and backflush of the medium inflow on dissolved oxygen concentration in anaerobic bioreactors. **(A)** Schematic representation of ‘bleed’ and ‘backflush’ to eliminate stagnant medium in the inlet tubing that had acquired oxygen by permeation through tubing during sequencing-batch reactor (SBR) experiments. The bleed operation disposes medium from tubing between medium reservoir and the sampling port. A separate ‘backflush’ operation uses overpressure in the reactor to push stagnant medium between reactor and sample point into the sample bottle. **(B)** A bioreactor assembled according to Protocol 2 was filled with tap water. Dissolved oxygen in the liquid phase was measured with a sensitive Hamilton VisiTrace Optical Trace DO 225 (Hamilton, Bonaduz GR, Switzerland) sensor equipped with an optical dissolved oxygen cap (L0-80) during an empty-refill sequence of the bioreactor with bleed, without (black line) and with the backflush operation (red line). Dissolved oxygen data were recorded with Android application ArcAir (Hamilton).

In SBR cultures, medium in the tubing between the medium reservoir and the reactor is stagnant in between empty-refill cycles. We observed that slow permeation through tubing caused entry of oxygen into this stagnant medium. To prevent entry of this oxygenated medium into the bioreactor, the ‘fill’ phase was preceded by pumping the first 10 mL of sterile medium into a dedicated sample bottle placed between the bioreactor and the medium pump. Subsequently, the medium pump was stopped and the mild overpressure in the reactor was used to also evacuate the tubing between the bioreactor and the sample bottle (Fig.   4 and Supplementary file S1) before refilling the reactor.

**Figure 5.**
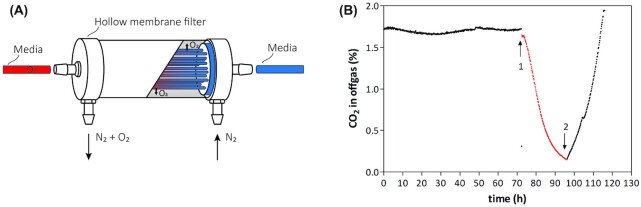
The effect of a membrane-contactor removing oxygen from the medium feed on cultures in chemostat. **(A)** Schematic representation of the PDMSXA-1000 membrane module (PermSelect, Ann Arbor, MI). Ingoing medium contaminated with oxygen (red) due to permeation through tubing, is stripped from oxygen with 5.0 quality nitrogen gas (Linde, Schiedam, The Netherlands) and the resulting anaerobic medium (blue) enters the bioreactor. **(B)***Saccharomyces cerevisiae* CEN.PK113-7D was grown in an anaerobic chemostat culture, as described in Protocol 2. Cultures were grown at a dilution rate of 0.10/h on synthetic medium with urea as nitrogen source (Luttik *et al*. 2000) and 20 g/ L glucose without supplementation of a source of either UFAs or sterols. Growth was monitored by on-line analysis of the CO_2_ concentration in the off gas. After 70 h, when the steady-state residual glucose concentration was 5.0 mM (indicated by Arrow 1), the medium inlet was rerouted through the membrane module, resulting in a washout (Red line). After 96 h, when the residual glucose concentration had increased to 66 mM, the medium flow was restored to the original situation state (Arrow 2).

Even when a glass medium reservoir is continuously flushed with high-purity (N6) N_2_, small amounts of oxygen were found to enter bioreactors with the ingoing medium flow. This problem was most pronounced in chemostat cultures, into which medium is slowly pumped from the reservoir to the reactor and, even when using tubing with a low oxygen permeability, may become contaminated with oxygen due to permeation. Visser *et al*. (1990) identified this mechanism as a major source of oxygen entry and placed a separate sterile, nitrogen-sparged, stirred bioreactor just in front of the actual chemostat bioreactor. We recently found that small, autoclavable membrane-contactor modules commonly used for gas exchange (Orgill *et al*. 2013; Bakonyi *et al*. 2015; Engler *et al*. 2018) are extremely efficient, affordable and practical devices for deoxygenating the medium feed of continuous-cultivation systems (Fig. 5). When a membrane-contactor module was placed near the medium entry point of bioreactors and connected to a flow of nitrogen (N5.5), *S. cerevisiae* chemostat cultures grown on glucose synthetic medium without the anaerobic growth factors ergosterol and Tween 80 completely washed out. This result marks a strong improvement on previous systems in which, under the same conditions, oxygen entry invariably led to reduced but significant steady-state biomass concentrations (da Costa *et al*. 2018, 2019; Dekker *et al*. 2021).

## CONCLUSIONS AND PROTOCOLS

Many aspects of the anaerobic physiology of yeasts can be studied in bioreactors or shake-flasks without requiring the extreme measures discussed above. For example, energy coupling and product yields on glucose can be reliably measured in bioreactors sparged with nitrogen (N5.5) gas and equipped with Norprene tubing (Weusthuis *et al*. 1994; Mans *et al*. 2017). Experimental design for investigations into the small oxygen requirements of yeasts for biosynthetic reactions starts with the sober realization that complete elimination of oxygen from bench-top set-ups, and even from anaerobic chambers, is virtually impossible. Unless elaborate measures are taken, such as pure-nitrogen sparging of cultures inside an anaerobic chamber (Waldbauer, Newman and Summons 2011), the key challenge is to consistently and verifiably reduce oxygen entry to levels that allow for meaningful experiments. Whenever possible, inclusion of oxygen-dependent control strains and/or verification of conclusions by genetic modification, is therefore essential.

In our research on anaerobic cultivation of yeasts, we identified carry-over of anaerobic growth factors from aerobically grown pre-cultures, frequent use of air locks in anaerobic chambers and oxygen permeation through bioreactor tubing as key points of attention. Serial transfer was found to be an essential and reliable approach to prevent misinterpretation of results caused by intracellular reserves of anaerobic growth factors. Permeation of oxygen diffusion through tubing was found to be particularly relevant for stagnant medium in tubing during SBR cultivation and during slow supply of medium during continuous operation. Use of membrane-contactor modules is a simple and promising approach to address the latter problem.

Below, two basic protocols for anaerobic cultivation of yeasts in anaerobic chambers and bioreactors are presented, along with comments that describe or explain specific points of attention. Clearly, differences in equipment, lab infrastructure and research goals may require other or additional measures. The main goal of these protocols is therefore not to provide a generally applicable manual, but to alert colleagues to potential pitfalls and possible solutions, and thereby aid them in interpreting published studies and in setting up anaerobic yeast cultivation experiments in their laboratories.

## PROTOCOL 1: ANAEROBIC CHAMBER

The following step-by-step description of growth experiments in an anaerobic chamber complements the Materials and Methods section of a publication in which we used this protocol to study UFA-independent anaerobic growth of *S. cerevisiae* (Dekker *et al*. 2019).

1. Place an orbital shaker platform and a small spectrophotometer, both cleaned with suitable disinfectant, in the previously cleaned workspace of the anaerobic chamber.
2. Generate an anaerobic environment in the workspace of the chamber according to manufacturer's protocol, and check its anaerobicity with a test culture (see Note 1).
3. Thoroughly clean all equipment and materials that will be introduced into the workspace with a suitable disinfectant (see Note 2).
4. Place cleaned containers/flasks containing sterile pipette tips, spectrophotometer cuvettes, demineralized water, concentrated solutions of anaerobic growth factors of interest, along with shake flasks filled with relevant sterile media, calibrated pipettes, a closable waste bin and any other required materials in the air lock of the anaerobic chamber, together with a freshly activated catalyst cartridge (see Note 3). Fill up the void volume of the air lock with oxygen-impermeable materials.
5. Perform at least four vacuum/purge cycles of the air lock, including two with hydrogen-containing gas, to aid removal of oxygen by the Pd catalyst cartridge before opening the inner door of the air lock.
6. Move all required materials into the workspace of the anaerobic chamber (see Note 4).
7. Repeat steps 3–6 until all required materials are in the workspace. Make sure all materials are in the workspace 2 days before starting an experiment, to allow oxygen to be removed, especially from liquid media.
8. Grow the strains of interest and control strains (see Note 1) in an aerobic incubator to mid-exponential phase.
9. Prepare inocula for anaerobic experiments in small volumes (typically up to 2% of final culture volume, to minimize introduction of dissolved oxygen into anaerobic pre-cultures via aerobically pre-grown inocula). Concentrate samples if necessary.
10. Transfer inocula into the workspace as described in steps 3–6.
11. Inoculate (see Note 5) the anaerobic pre-cultures at the desired optical densities.
12. Monitor optical density of the anaerobic pre-cultures over time with a spectrophotometer placed in the workspace (see Note 6). Next steps depend on the growth profile (see Fig. 2):
  - Growth is observed (Fig. 2A and B; the culture uses intracellular reserves of anaerobic growth factors or can grow anaerobically in the absence of the growth factor of interest). Continue with step 13.
  - No growth is observed (Fig. 2C and D; the yeast cannot use intracellular reserves, has additional oxygen requirements, is unable to take up the growth factor of interest or the culture is no longer viable). Continue with step 14.
13. When the optical density no longer increases, transfer a small aliquot of the culture to separate flasks with anaerobic media, supplemented with various combinations of the anaerobic growth factor(s) of interest. Monitor growth as described above until the end of the experiment. Continue with step 15.
14. Add an appropriate volume of the concentrated solution of the anaerobic growth factor(s) of interest to the culture:
  - Growth is observed (Fig. 2C; the yeast grows anaerobically when provided with this anaerobic growth factor).
  - No growth is observed (Fig. 2D; the yeast has additional oxygen requirements, is unable to take up the growth factor of interest or the culture is no longer viable).
15. After terminating the anaerobic growth chamber experiment, move cultures that did not grow out of the anaerobic chamber and incubate them aerobically for a provisional indication of culture viability.

## PROTOCOL 2: ANAEROBIC CULTIVATION IN BIOREACTORS

This protocol outlines key steps for anaerobic batch, sequential-batch and chemostat cultivation in bioreactors. Information on equipment and materials used in our laboratory can be found in recent publications (Juergens *et al*. 2018; Dekker et al. 2019, 2021; see Supplementary file S2).

## BIOREACTOR BATCH CULTIVATION

Steps 1–9 describe anaerobic bioreactor batch experiments. Since sequencing-batch and chemostat experiments are usually started as batch cultures, these steps also apply for those modes of cultivation.

1. Before assembling a bioreactor set-up, thoroughly check all tubing, seals, septa and O-rings for wear or damage and replace them when necessary. Minimize and standardize length of tubing for replicate bioreactors (see Note 7).
2. Clamp all tubing, apply an 0.4 bar overpressure and monitor pressure for at least 15 min. If a pressure drop is observed, submerge the bioreactor in water to identify the leak. Prior to autoclaving the bioreactor, remove clamps to ensure gas exchange is possible.
3. Aseptically fill the autoclaved bioreactor with sterile medium to the intended working volume minus the volume of the inoculum.
4. Activate gas analysis equipment, mass flow controllers, pressure valves and equipment used for control of process parameters (e.g. temperature and stirrer speed).
5. Sparge medium in the bioreactor and set overpressure at 0.2 bar (see Note 8). Continue sparging for at least 1 h with high-purity nitrogen (N6 or above) at 0.5 L N_2_/(L working volume)/min (see Note 9).
6. Release overpressure, then stop gas flow and inoculate the bioreactor.
7. Redirect inlet nitrogen stream through bioreactor headspace, stop sparging of N_2_ through culture broth, and reapply 0.2 bar overpressure.
8. Clamp all tubing that is not actively used during the growth experiment as close as possible to the bioreactor (see Note 10).
9. Use the 0.2 bar overpressure for aseptic sampling and take a pre-sample with each sample to discard any stagnant culture from tubing.
  For further operation as a sequential batch reactor experiment, continue at step 10. For further operation as a chemostat, continue until step 11 and then proceed to step 21.
10. Assemble glass medium reservoir using oxygen impermeable tubing, O-rings and include sparging equipment (e.g. air stone).
11. Aseptically connect sterile medium reservoir and effluent to bioreactor influent and effluent, respectively, via peristaltic pumps.
12. Under the chosen process conditions, aseptically adjust level sensor to the desired working volume of the subsequent batch-cultivation cycles (see Note 11). Connect the level sensor to influent pump, to stop pumping upon contact.
13. Leave 0.2 – 1.0% of the working volume set in step 12 after the emptying phase. The ratio of residual over the working volume determines the number of generations in each batch-cultivation cycle (see Note 12).
14. Vigorously sparge medium reservoir with nitrogen (N5.5) gas for at least 1 h before use and continue sparging until refilling of bioreactor is complete (see Note 9).
15. Empty the bioreactor by manually or automatically switching on the effluent pump (see Note 13).
16. Prior to refilling the bioreactor, take a sample of 10 mL from the medium inlet to discard stagnant medium from the tubing, using a sampling port close to the bioreactor lid. Use the 0.2 bar overpressure to backflush the medium in between the sampling port and the bioreactor (Fig. 4A)
17. Activate the influent pump to start filling the bioreactor and switch the gas inflow from headspace to sparging to minimize oxygen entry via medium inflow.
18. Inflow of medium will automatically stop when the medium reaches the electrical level sensor. Gas inflow can be reverted to headspace (see Note 14).
19. Wait until the culture has depleted the limiting medium component, usually indicated by a decrease of the CO_2_ concentration in the exhaust gas (see Note 15).
20. To initiate a subsequent empty refill cycle, repeat steps 14–19 for another empty refill cycle.
  After the anaerobic batch reactor experiment (steps 1–9), connect the medium vessel (steps 10 and 11) and continue with the next steps for an anaerobic chemostat experiment.
21. Connect the level sensor to the effluent pump, or ‘pump on contact’ mode (see Note 16).
22. Set the influent pump to a rate corresponding to the desired dilution rate of the continuous culture. Adjust level sensor if required. A culture is considered to be in a steady state when during at least five volume changes the culture parameters and physiology did not differ more than a predefined margin over three subsequent samples taken at least one volume change apart (see Note 17)
23. When steady-state conditions have been reached, start sampling for steady state characterization. The experiment can be stopped, the overpressure released and the broth weighed to determine the actual working volume.

## NOTES ANAEROBIC CHAMBER

**Note 1**. As controls in anaerobic growth chamber experiments, we routinely include cultures of *S. cerevisiae* CEN.PK113-7D on glucose synthetic medium with and without Tween 80 and ergosterol. If, after a first anaerobic growth cycle to deplete intracellular reserves, sustained growth is observed on glucose synthetic medium without Tween 80 and ergosterol, this is a strong indication for the presence of oxygen in the workspace. See Fig. 2B for representative results of negative control cultures in which only a very slow increase of optical density is observed.

**Note 2**. Because no fire/Bunsen burner can be used, strict measures are needed to reduce the risk of contamination. Surfaces and equipment must be regularly disinfected with 70% ethanol or other suitable disinfectants. Oxygen permeability of butyl rubber increases upon repeated or prolonged exposure to ethanol. Therefore, avoid spilling of ethanol on gloves while cleaning. We recommend using sterile pipette tips equipped with filters, and to clean pipettes with ethanol in between sampling of different cultures. Even when taking extensive precautions, be alert to the possibility of (cross) contamination of cultures.

**Note 3**. The Pd catalyst cartridge aids the removal of traces of oxygen by catalyzing oxidation of hydrogen in the anaerobic gas mixture (up to 5% H_2_, 5 – 10% CO_2_ and N_2_). Because this process generates water, the catalyst needs to be regularly reactivated by dry heating. Consult manufacturer's instructions for frequency of recycling, but be aware of trade-offs related to frequent use of the air lock. We limit use of the air lock to twice a week, despite the manufacturer's advice to re-activate the catalyst daily. This limited use of the air lock requires careful scheduling of entry and removal of materials.

**Note 4**. Frequent use of the air lock is a main cause of oxygen contamination in anaerobic chamber experiments (Fig. 2). Preferably, materials introduced via the air lock should be accompanied by an activated catalyst cartridge and incubated in the air lock for at least 30 min to reduce oxygen entry.

**Note 5**. In the anaerobic pre-culture, intracellular reserves of anaerobic growth factors should be depleted. To prevent premature depletion of glucose we recommend using a high initial concentration (5% w/v) for this pre-culture.

**Note 6**. Working with gloves complicates taking notes of measurements made within the anaerobic workspace. A small voice recorder attached to the anaerobic chamber facilitates recording of culture number, time and optical density.

## NOTES BIOREACTOR

**Note 7**. To minimize oxygen entry, several modifications were made to our standard bioreactor setups. Silicone sealing rings in the headplate were replaced with less oxygen-permeable Viton rings (Eriks, Rotterdam, NL). Nylon tubing was used for non-aseptic parts of the gas supply and the length of the gas line from cylinder to bioreactor was minimized. When near-empty gas cylinders were replaced for full ones, the gas supply line was purged before reconnection. Where possible, plastic parts (e.g. tubing connectors and sterile cotton-wool filter canisters in gas lines) were replaced with stainless steel parts.

**Note 8**. Throughout cultivation, experimenters should be aware that the bioreactor is operated under overpressure, which pushes broth out of the bioreactor when the effluent line is opened. In addition, if the gas flow through the bioreactor is interrupted while sparging, broth can be pushed into the gas inlet. Install liquid traps to protect expensive mass flow controllers and be mindful to always release the overpressure from bioreactor before changing the gas flow.

**Note 9**. During sparging with nitrogen (N5.5 or above), the dissolved oxygen (DO) concentration asymptotically approaches zero. Depending on the volumetric mass-transfer coefficient (k_L_a), 90% of the oxygen is usually already removed within 1 h. Sparging time prior to the experiment can be increased, but near-complete removal of oxygen may take several hours.

**Note 10**. Even use of highly oxygen-impermeable tubing (e.g. Fluran F-5500-A) does not completely eliminate oxygen permeation through tubing. Clamping tubes close to the bioreactor head plate helps to minimize this mode of oxygen entry.

**Note 11**. Adjusting the level sensor towards the top of the turbulent liquid level, while the bioreactor is operating at its mixing, gassing and temperature setpoints, ensures that a correct and constant working volume is maintained. To prevent adjustments of the level sensor from compromising aseptic conditions, 70% ethanol can be applied to the level sensor seal.

**Note 12**. When biomass concentrations at the end of each batch cultivation cycle are the same, the number of generations per cycle roughly corresponds to the number of doublings of the culture volume (e.g. leaving 25 mL of broth after the emptying phase in an SBR that is subsequently refilled to a working volume of 1600 mL will result in six generations per SBR cycle).

**Note 13**. It is important to empty the bioreactor as fast as possible. During the majority of the emptying process the broth can still be mixed but as the liquid-gas interface drops below the impellers, the broth becomes stagnant which may lead to sedimentation of yeast to the bottom of the bioreactor. Cells at the bottom of the bioreactor are not removed via the effluent pipe as it is not located at the absolute bottom of the bioreactor, thus selecting for fast sedimenting yeast and reducing the number of generations per cycle (Oud *et al*. 2013).

**Note 14**. After re-filling of the bioreactor is initiated, we recommend to sparge the broth with high-purity nitrogen (N6) to rapidly ‘strip’ any remaining oxygen in the medium. After filling is completed, we redirect nitrogen supply through the headspace to minimize transfer of traces of oxygen in the nitrogen gas into the liquid phase (see text and Fig. 3).

**Note 15**. When sugar is the first nutrient to be depleted, this usually coincides with a sharp decline of the CO_2_ concentration in the exhaust gas. Continuous monitoring of the CO_2_ concentration in the exhaust gas is then a useful trigger mechanism for initiating a new batch. If another nutrient becomes limiting first, this may not lead to an immediate decrease of the CO_2_ output, and other trigger mechanisms must be employed.

**Note 16**. The control loop connected to the level sensor and pump operates in opposite modes in chemostat or sequential batch reactor experiment. In chemostats, contact of the broth with the level sensor is used as a signal to start the effluent pump and thereby keep the volume of the broth constant over time. In contrast, during sequential batch reactor experiments, pumping of fresh medium is terminated upon contact when the desired working volume is reached.

**Note 17**. Achieving a steady state is an asymptotic process, during which adjustments to the culture introduce undesired dynamics. Adjustments of the cultivation conditions should therefore be performed directly after the batch phase or early on in the chemostat experiment. Steady state is assumed when at least five volume changes have occurred after the last change in growth conditions and, moreover, the biomass concentration, the concentration of the growth limiting nutrient and important biomass-specific production and consumption rates differ by less than a predefined margin (e.g. 1, 2 or 5%, depending on the experimental goals) for a further two consecutive volume changes.

## ACKNOWLEDGEMENTS

We thank our colleagues at the Indusrial Microbiology section for support and stimulating discussions.

## Supplementary Material

foab035_Supplemental_File
