# Supplementary material for: Critical parameters and procedures for anaerobic cultivation of yeasts in bioreactors and anaerobic chambers
Source: FEMS Yeast Res. 2021 Jun 8;21(5):foab035. doi: 10.1093/femsyr/foab035 (PMC8216787; doi:10.1093/femsyr/foab035)

**Supplemental material for:**

**Critical parameters and procedures for anaerobic cultivation of yeasts in bioreactors and anaerobic chambers**

Christiaan Mooiman, Jonna Bouwknegt, Wijb J.C. Dekker, Sanne J. Wiersma, Raúl A. Ortiz-Merino, Erik de Hulster and Jack T. Pronk*

Delft University of Technology, Department of Biotechnology, Van der Maasweg 9, 2629 HZ Delft, The Netherlands

^*^Corresponding author: Jack T. Pronk; e-mail j.t.pronk@tudelft.nl; phone +31 15 2783214; Address: Delft University of Technology, Department of Biotechnology, Van der Maasweg 9, 2629 HZ, Delft, The Netherlands

**
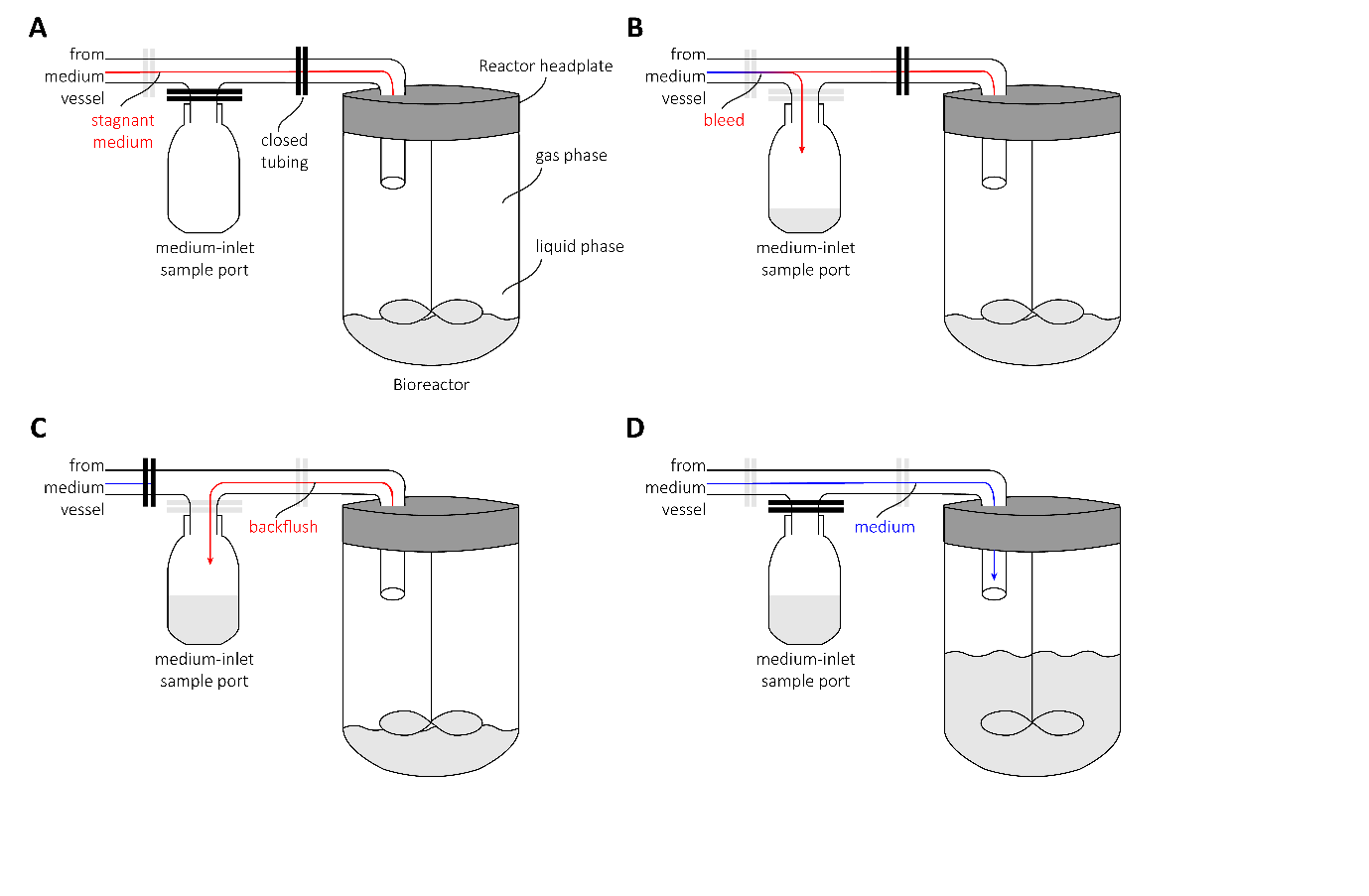
Figure S1: Schematic illustration of bleed and backflush operations to refill anaerobic bioreactors with deoxygenated medium.** (**A**) The stagnant medium in the medium inlet line becomes contaminated with oxygen (depicted in red) due to slow permeation through the tubing. (**B**) The medium pump is activated and used to pump stagnant medium into a sample bottle placed between the medium pump and the bioreactor, until stagnant medium in the tubing is completely replaced by deoxygenated medium (blue) from the medium vessel. (**C**) Stagnant medium between the sample bottle and the bioreactor is carefully flushed back from the tubing using the pressure of the bioreactor gas phase into the sample bottle. (**D**) The sample bottle is closed and deoxygenated medium is pumped into the bioreactor.

**Figure S2: Schematic process gas flow diagram of bioreactor set-up for anaerobic and microaerobic SBR and chemostat cultivation.**


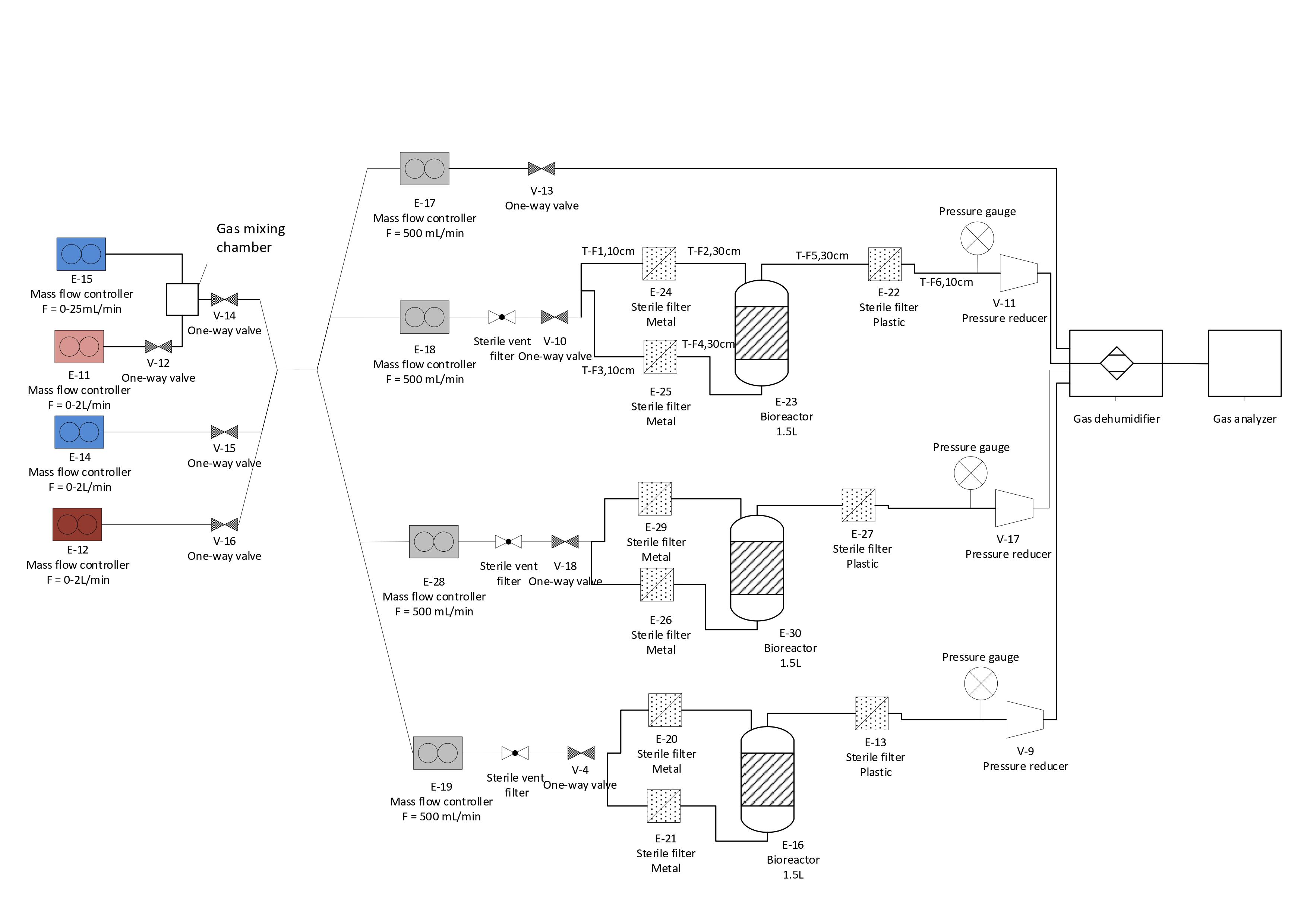


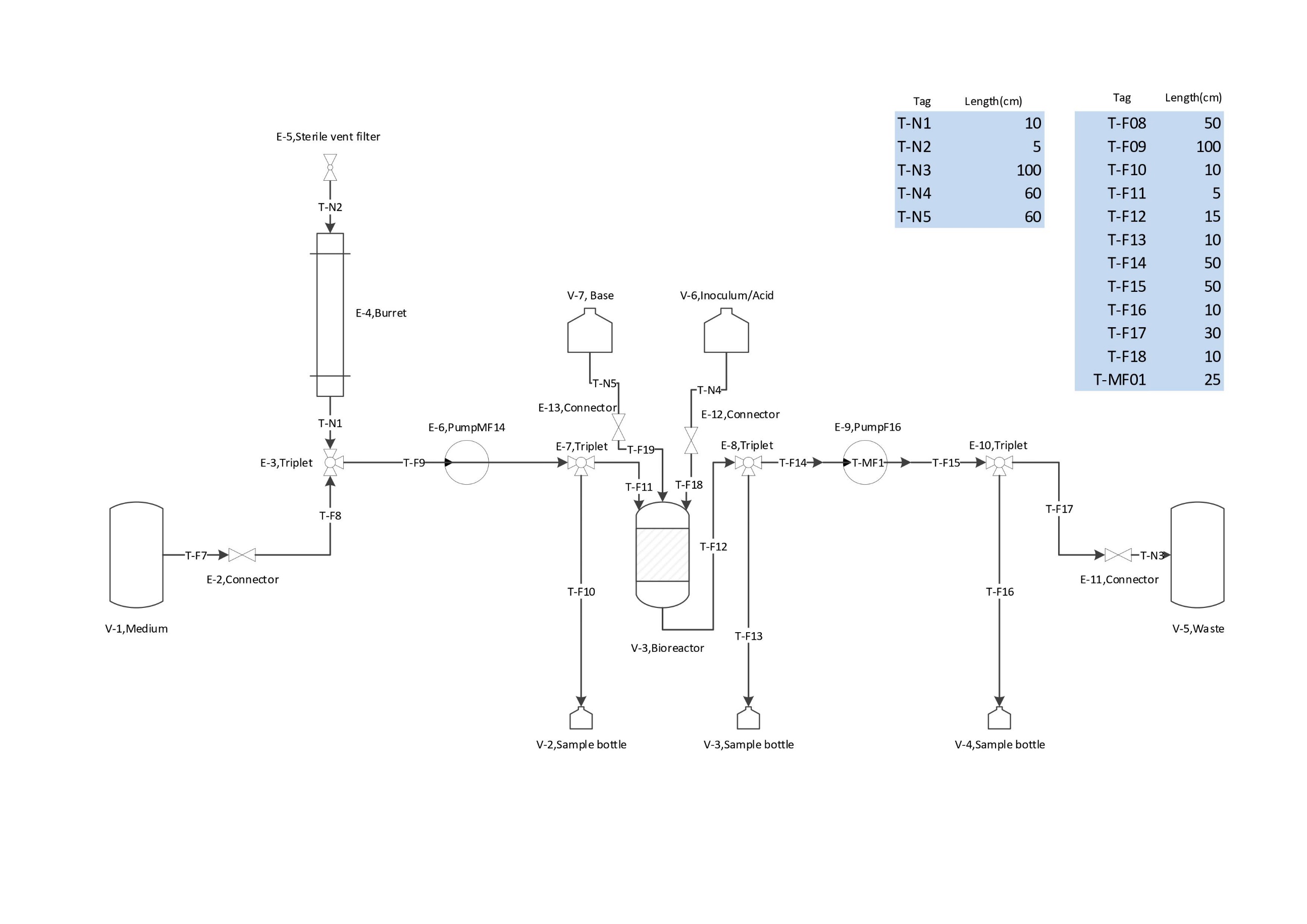

Supplement: foab035_Supplemental_File [file foab035_supplemental_file.docx]
